# Imaging of intermittent lipid-receptor interactions reflects changes in live cell membranes upon agonist-receptor binding

**DOI:** 10.1038/s41598-019-54625-w

**Published:** 2019-12-02

**Authors:** Johan Tornmalm, Joachim Piguet, Volodymyr Chmyrov, Jerker Widengren

**Affiliations:** Experimental Biomolecular Physics, KTH, 10691 Stockholm, Sweden

**Keywords:** Wide-field fluorescence microscopy, Biological fluorescence, Cellular imaging

## Abstract

Protein-lipid interactions in cellular membranes modulate central cellular functions, are often transient in character, but occur too intermittently to be readily observable. We introduce transient state imaging (TRAST), combining sensitive fluorescence detection of fluorophore markers with monitoring of their dark triplet state transitions, allowing imaging of such protein-lipid interactions. We first determined the dark state kinetics of the biomembrane fluorophore 7-nitrobenz-2-oxa-1,3-diazole-4-yl (NBD) in lipid vesicles, and how its triplet state is quenched by spin-labels in the same membranes. We then monitored collisional quenching of NBD-lipid derivatives by spin-labelled stearic acids in live cell plasma membranes, and of NBD-lipid derivatives by spin-labelled G-Protein Coupled Receptors (GPCRs). We could then resolve transient interactions between the GPCRs and different lipids, how these interactions changed upon GPCR activation, thereby demonstrating a widely applicable means to image and characterize transient molecular interactions in live cell membranes in general, not within reach via traditional fluorescence readouts.

## Introduction

G-protein coupled receptors (GPCRs) represent a super-family of membrane-embedded proteins, consisting of seven transmembrane helices and an additional intracellular helix. GPCRs represent the largest class of membrane proteins involved in signal transduction across cell membranes and are major targets in the development of new drug candidates^1^. The details of how GPCRs can translate extracellular signals into specific intracellular responses are intricate. Although many underlying mechanisms have been clarified by biochemical and biophysical investigations, additional mechanisms remain to be revealed to explain the broad, yet precise and efficient, intracellular actions of GPCRs. Increasing evidence suggest that interactions with membrane lipids play a major role in modulating the function of GPCRs^2–4^. Such interactions include binding of specific lipids, in particular cholesterol, to specific sites on the GPCRs, formation of domains with specific lipid compositions around the GPCRs^5^, lipid selectivity to the protein^6^, or localized deformations of the membrane bilayer around the proteins^7^. However, these interactions remain insufficiently understood. They are largely transient in character, typically take place on microsecond time scales, and on a spatial scale of nanometers, and are therefore challenging to characterize by experimental approaches^8^. Expanded abilities to analyze GPCR-lipid interactions will provide key information for understanding GPCR dynamics, activation and signaling, which in turn will open new possibilities for selective drug development^9^.

Fluorescence methods offer a very high sensitivity and high time resolution compared to other biophysical techniques used to study biological membranes, such as NMR, EPR and FTIR, and allow readouts in systems of varying complexity, up to the level of living cells and tissue^10^. For these reasons, fluorescence methods are also the most extensively used for dynamics and interaction studies of GPCRs and other membrane proteins. Molecular co-localizations in membranes can be determined, using statistical analyses of dual color fluorescence microscopy images^11^, Förster resonance energy transfer (FRET)^12,13^, and bimolecular fluorescence complementation (BiFC) assays^14^. Interactions can also be studied via altered diffusion behavior of the interacting molecules in the membranes using several different fluorescence techniques, including fluorescence correlation spectroscopy (FCS), fluorescence recovery after photobleaching (FRAP), and single-particle tracking (SPT) (see refs. ^15–17^ for reviews). By dual-color Fluorescence Cross-Correlation Spectroscopy (FCCS)^18,19^, interactions between two molecular species, with fluorescence emission in two spectrally separated bands can be analyzed in parallel with their diffusion behavior. The interaction is then characterized not only by mere co-localization of the two species upon binding to each other, but also by their concerted movement into and out of a diffraction-limited detection volume. In general however, these methods primarily address self-diffusion and are not directly suited to monitor transient molecular interactions in the membranes^20^. For GPCRs, such interactions seem central to many of their working mechanisms.

Transient interactions between molecules in membranes, as those taking place between GPCRs and lipids in cell membranes, have mainly been studied by luminescence quenching, with luminescence probes and quencher molecules labeled to interaction partners in the membranes. With fluorescence emission used as readout in such quenching studies, an excellent detection sensitivity can be obtained. However, most fluorophores have excited state lifetimes in the range of nanoseconds, while bimolecular diffusion-mediated processes in biological membranes typically take place on three to six orders of magnitude longer time scales. Fluorescence lifetimes are too short to allow diffusion-controlled collisional encounters with quenchers to significantly affect the fluorophore emission. Fluorescent probes with longer fluorescence lifetimes are rare, in particular if they both need to have a high fluorescence brightness and to be efficiently quenched by contact^21^. Phosphorescence labels are considerably more long-lived, and from this point of view more suitable for bimolecular reaction studies in membranes. On the other hand, as a consequence of their long emission lifetimes, the phosphorescent triplet states of these probes are also more susceptible to quenching by oxygen and trace impurities. By this quenching the phosphorescence lifetime is not only shortened, it also makes the signal practically undetectable. The use of phosphorescence as a readout for molecular encounter studies in membranes is thus largely restricted to deoxygenated, carefully prepared samples. Alternatively, larger, shielded, and therefore less environment sensitive probes have to be used, with limited applicability. As a way to overcome these drawbacks, quenching of long-lived triplet states of fluorophores can be monitored via fluorescence, offering a signal many orders of magnitude stronger than phosphorescence emission. In FCS measurements, the kinetics of triplet^22^ and other dark transient states^23–25^ of fluorophores can be monitored via the intensity fluctuations of their emitted fluorescence. Thereby, high detection sensitivities can be combined with the benefit of the long lifetime and environmental sensitivity of the triplet state. In previous work, we showed how FCS measurements, monitoring fluorescence intensity fluctuations resulting from transitions between singlet and triplet states in membrane-bound fluorophores, can be used to quantify quenching of their triplet states by spin labels in the membranes^26^. Thereby, molecular encounters between the fluorophores and spin labels in the membranes can be monitored at frequencies, at which practically no quenching of the fluorescence intensity occurs. However, since FCS relies on detection of stochastic fluorescence intensity fluctuations originating from dynamic events of individual molecules, the application of this approach is limited to single-molecule detection conditions. Moreover, high brightness fluorophores in nano-molar concentrations are needed, and have to be detected in samples with low background and at high time resolution.

In this work, we apply transient state (TRAST) imaging to monitor the quenching of dark transient states of a membrane fluorophore by spin labels in the same membrane, as a means to characterize low-frequency, intermittent interactions between molecules in cellular membranes. In TRAST, the plain, time-averaged fluorescence intensity from a sample is recorded, when subject to time-modulated excitation^27–29^. By measuring how the average fluorescence intensity varies with the excitation modulation characteristics, full kinetic information about photo-induced, non-fluorescent transient states of the fluorophores in the sample can be obtained. Like FCS, TRAST measurements combine the sensitivity of fluorescence detection with the environmental sensitivity of the long-lived triplet and other dark transient states that can be monitored. In contrast to FCS however, no particular time resolution in the fluorescence detection is required, and since TRAST measurements do not require single-molecule detection conditions, they can be applied to monitor long-lived dark transient states of fluorescent molecules in a wide range of biological samples^29,30^.

Here, we applied TRAST to determine the dark state kinetics of 7-nitrobenz-2-oxa-1,3-diazole-4-yl (NBD). NBD is a small fluorophore, with limited steric effects when used as a label, and is widely used for membrane studies^31^. We show that the transient state kinetics of NBD, when labelled to lipids in the membranes of small unilamellar vesicles (SUVs) and live cells, are strongly influenced by presence of spin-labels in the same membranes. At the same time, by regular recording, practically no effects were observed in the fluorescence intensity levels from the NBD probe molecules. We then used the TRAST approach to follow the interaction between a spin-labeled GPCR (the neurokinin 1 receptor, NK1R) and NBD-labeled lipids in the plasma membranes of live cells. NK1R is involved in several physiological and patho-physiological functions and is an important therapeutic target^32^. In its resting state, NK1R has been found to form clusters in the plasma membrane of 293 T cells^33^. After activation with its natural ligand, substance P (SP), the receptor localization then changes significantly^34^. This reorganization is principally the result of the fast internalization of activated receptors. We found that the transient state kinetics of the NBD labels differed significantly upon NK1R activation by its agonist SP. This reflects differences in the transient interactions between the GPCR and the labelled lipids, which could be attributed to modifications in the membrane environment upon activation. The observed changes could be monitored via the quenching rates, both on a whole-cell level, but also in a spatially resolved manner with sub-cellular resolution. This shows that the approach presented in this work can characterize and image transient interactions between GPCRs and molecules in their membrane environment, in a straightforward and widely applicable manner. Given the importance of GPCRs in cell biology and as drug targets, the central role of transient interactions in the membranes for modulating their functions, and that these interactions are very difficult to determine by other means, we believe that the presented approach will be useful in future GPCR research and drug development.

## Results

**Figure 1:**
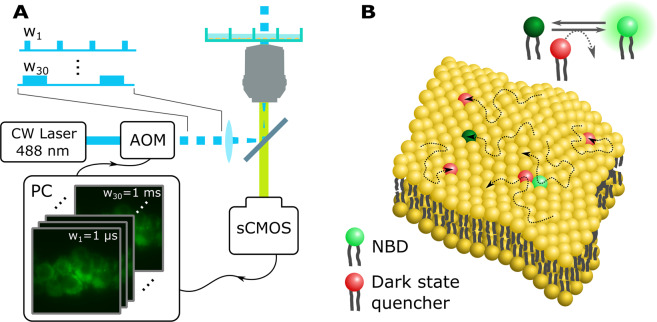
Membrane interaction assay. **(A)** Optical TRAST setup. The laser is modulated using an acousto-optic modulator (AOM) and defocused to produce a wide-field illumination of the sample (*ω*_0_ = 35 *μm*, 1/*e*^2^ radius). A stack of images acquired using different pulse durations, *w*, are converted to images of dark-state transition rates of the fluorophores in the sample. **(B)** With the NBD fluorophore and dark state quencher tagged to different lipids in a membrane, low-frequency collisional interactions between these lipids will be sufficient to quench the NBD dark state and can be observed in the recorded TRAST images.

An instrument for wide-field TRAST microscopy was established, as further described in the Methods section. We applied low duty cycle (η = 1%) rectangular excitation pulse trains with different excitation pulse durations, *w*, and recorded how the average fluorescence intensity from the sample during excitation, 〈*F*_*exc*_〉 (defined in Eqs. 2 and 3), varied with *w* (Fig. 1A). By this approach, we first quantified the photophysical properties of NBD, then investigated to what extent collision rates between the fluorescent probe (NBD) and triplet state quenchers (DOXYL/TEMPO), both labelled to lipids in the membranes of SUVs and then in live cell membranes, are reflected in the dark-state kinetics of NBD. The measurement strategy is further described in Fig. 1B.

### TRAST measurements on SUVs

#### Photophysical model for NBD

**Figure 2:**
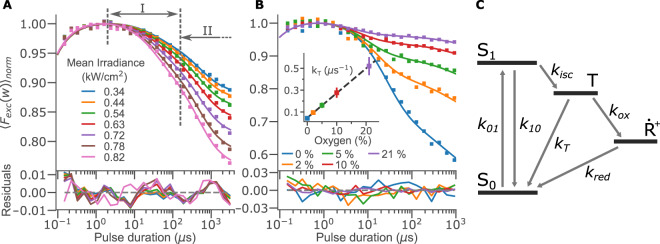
NBD photophysics. (**A**) TRAST curves from NBD-PC12 embedded in POPC SUVs in deoxygenated PBS buffer, measured under different excitation irradiances. The time scales for T and ${\dot{R}}^{+}$ state relaxation are denoted I and II, respectively. **(B)** TRAST curves from the same NBD-PC12 sample, measured at 3.2 kW/cm^2^, with varying concentrations of atmospheric oxygen. (In this case, a higher excitation irradiance than in (A) was used to induce a well observable triplet state build-up also under air-saturated conditions ([O_2_] = 21%)). The fitted triplet quenching rates are shown in the inset. **(C)** Electronic state model used to fit the NBD TRAST data. Excitation takes place from the singlet ground state (*S*_0_) to the first excited singlet state (*S*_1_) at a rate *k*_01_. *S*_1_ can either return to *S*_0_ at a rate *k*_10_ or undergo intersystem crossing at a rate *k*_*isc*_ to the triplet state (*T*). The triplet state has two deactivation pathways: to *S*_0_ via the triplet relaxation rate *k*_*T*_ and to a long-lived radical state (${\dot{R}}^{+}$) through the oxidation rate *k*_*ox*_. The redox state is deactivated to *S*_0_ through reduction, with a rate *k*_*red*_.

To establish the measurement procedures and determine a photophysical model for the dark-state transitions of NBD under our experimental conditions, we quantified the dark-state kinetics of NBD-PC12 lipid derivatives embedded in the membrane of freely diffusing POPC SUVs in an anoxic environment (see Methods and Materials). Given the Gaussian shape of the excitation laser focus (*ω*_0_ = 35 μm, 1/*e*^2^ radius), we could divide the fluorescence image into circular shells corresponding to regions within the same range of excitation intensities^35^. Thereby, we could record the fluorescence intensity at a range of excitation intensities from a single wide-field image. By systematically varying the rectangular excitation pulse durations while recording the average fluorescence intensities, 〈*F*_*exc*_(*w*)〉_*norm*_ (Eqs. 2 and 3), in each part of the image, TRAST curves at average excitation irradiances between 0.34 and 0.82 kW/cm^2^ were obtained (Fig. 2A). The recorded TRAST curves show two major relaxation processes, at ~20 μs and ~1 ms respectively (denoted I and II in Fig. 2A). The amplitudes of these dark states both increase with excitation irradiance, while their relaxation times decrease, consistent with a promoted build-up of dark triplet and redox states in the NBD fluorophores.

Next, we studied the effect of oxygen on the recorded TRAST curves of NBD. Figure 2B shows TRAST curves from the same NBD-PC12 probes in POPC SUVs, with the oxygen concentration in the measurement chamber, [O_2_], varied between 0% and 21%. Molecular oxygen is a potent triplet state quencher and is expected to significantly reduce both triplet state build-up and decay time^22^. In accordance with this, a high excitation irradiance (3.2 kW/cm^2^) was required to achieve sufficient contrast also under air-saturated conditions. Even so, only a very minor dark state build-up of a few percent was observed in this case, with a corresponding relaxation time of ~2 μs.

The excitation and oxygen dependence observed in Fig. 2A,B, is consistent with a photophysical model of NBD as outlined in Fig. 2C. Here, excitation from the singlet ground state (S_0_) to an excited singlet state (S_1_) leads to the population of a triplet state (T) and an oxidized state (${\dot{R}}^{+}$). In this model, fluorescence emission takes place from the excited S_1_ state, with a fluorescence lifetime of *τ*_*f*_ = 5.6 ns^36,37^, while the triplet state (with a relaxation time, *τ*_*T*_~1−100 μs, depending on oxygenation) and the photo-oxidized state (relaxation time, *τ*_*ox*_~1 ms) can both be considered non-luminescent. Given the different time scales for the relaxation of the S_1_, T and ${\dot{R}}^{+}$ states, it is difficult to kinetically distinguish if photo-oxidation can take place from S_1_, T, or from both states. The photo-oxidation quantum yield from T, with its three orders of magnitude longer lifetime than S_1_, is likely to be correspondingly higher. We therefore assume photo-oxidation to occur solely from this state, as also has been found for other fluorophores^38^.

As a further verification of the photophysical model, and to determine the transition rate parameters, we performed a global fit of the TRAST curves in Fig. 2A,B (see Methods and Materials). The excitation rate was calculated by *k*_01_ = *σ*⋅Φ_*exc*_, with an excitation cross section of *σ* = 8.6 ⋅ 10^−17^ cm^2^ ^36,39^ and where Φ_*exc*_ is the excitation photon flux. The triplet relaxation rate, *k*_*T*_, was allowed to vary freely for each curve, the intersystem crossing rate, *k*_*isc*_, was determined as a linear function of [O_2_], while *k*_*ox*_ and *k*_*red*_ were set as global variables. With photo-oxidation taking place from T only, the build-up of ${\dot{R}}^{+}$ is strongly dependent on the population of the triplet state. In comparison to the influence of [O_2_] on *k*_*T*_, any influence on *k*_*ox*_ and *k*_*red*_ from [O_2_] can be neglected. The S_1_ relaxation rate was determined to reproduce the experimental fluorescence lifetime under air-saturated conditions, *k*_10_ = 1/*τ*_*F*_−*k*_*isc*_. Overall, global fitting based on the photophysical model in Fig. 2C could well reproduce the experimental TRAST curves in Fig. 2A,B. The freely fitted triplet state decay rates are shown in the inset of Fig. 2B, which together with a linear fit gives *k*_*T*_ = 0.047 μs^−1^ + 1.7 μs^−1^ mM^−1^ [O_2_]. All fitted rate parameters are summarized in Table 1. The fitted bimolecular interaction rates with oxygen are similar for both *k*_*T*_ and *k*_*isc*_. They are of the same order of magnitude as typical diffusion-limited reaction rates for small molecules in water, and also match oxygen quenching rates determined for other organic fluorophores in aqueous solutions^38,40^.

**Table 1:** Photophysical rate parameters, together with 95% confidence intervals, determined for NBD-PC12 in POPC SUVs using TRAST.

	Base rate *μs*^−1^	O_2_ *μs*^−1^ mM^−1^	16-DOXYL *μs*^−1^ (*mol*%)^−1^	TEMPO-PC *μs*^−1^ (*mol*%)^−1^
*k*_*isc*_	5.7 ± 0.7	1.8 ± 0.3	1.4 ± 0.2	1.0 ± 0.2
*k*_*T*_	0.041 ± 0.007	1.6 ± 0.2	0.11 ± 0.02	0.22 ± 0.04
*k*_*ox*_	0.0007^*a*^			
*k*_*red*_	0.0005^*a*^			

The confidence intervals consider both the global fitting of the TRAST curves as well as the subsequent maximum likelihood estimation of the bimolecular quenching coefficients.

^a^Determined within one order of magnitude.

#### Collisional quenching of NBD derived lipids by spin labels

**Figure 3:**
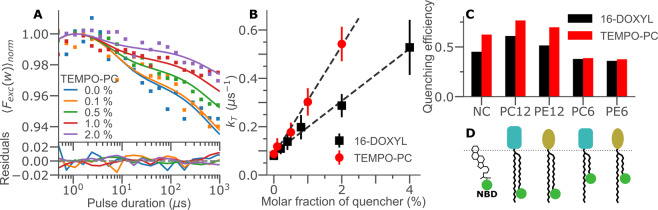
Spin-label quenching of the NBD triplet state. (**A)** TRAST curves of NBD-PC12 in POPC vesicles with varying molar fractions of TEMPO-PC. **(B)** Fitted triplet relaxation rates as a function of TEMPO (red circles, data from (A)) and of 16-DOXYL (black squares, data in SI). **(C)** Triplet state quenching efficiency (Φ_*Q*_, see Eq. 1), for different NBD probes, given by the difference in dark-state build-up in NBD in the absence and presence of 1% molar fraction of either TEMPO or 16-DOXYL as quencher. **(D)** Illustration of the different NBD positions investigated in (C).

Next, we investigated how the dark states of NBD are affected by triplet quenchers. Two common spin labels were used; 16-DOXYL stearic acid, containing an oxazolidinyloxy group on the 16th carbon of its aliphatic chain, positioning the triplet-quenching free radical deep in the hydrophobic core of the bilayer, and TEMPO-PC, containing a similar free radical on the lipid head group, locating the quencher to the surface of the membrane. To reduce competitive triplet state quenching by molecular oxygen, while still allowing live cell measurements to be performed with the same conditions later on, we performed these experiments in an atmosphere of 2% [O_2_]. For the same reason, the mean excitation irradiance in the experiments was set to 0.48 kW/cm^2^. This corresponds to a total light dose below 0.5 kJ/cm^2^ in all regions of the laser beam, for cells and SUVs alike, thereby not exceeding excitation doses where phototoxic effects on cells would occur^41^. Figure 3A shows TRAST curves recorded from SUVs containing NBD-PC12 and with varying molar fractions of TEMPO-PC added to the lipid membranes of the SUVs. With increasing TEMPO-PC concentrations, we observed a clear reduction in both the triplet amplitudes and the relaxation times in the recorded curves. Similar TRAST curves were also obtained when 16-DOXYL was used as a quencher, and with the same trends observed in the amplitude and relaxation times upon increasing 16-DOXYL concentrations (see Supplementary Fig. S1).

Global rate parameters were fitted based on the same model and fitting procedure as before. The resulting *k*_*T*_ rates for both TEMPO-PC and 16-DOXYL are shown in Fig. 3B. In agreement with a bimolecular interaction, *k*_*T*_ showed a linear dependence on the concentration of TEMPO-PC and 16-DOXYL in the membranes. Fitting of the determined *k*_*T*_ values to a linear model yielded quenching coefficients of 0.22 μs^−1^ and 0.11 μs^−1^ per molar percentage for TEMPO-PC and 16-DOXYL, respectively. Similarly, a linear increase in *k*_*isc*_ with increasing spin-label concentrations was also observed, see Table 1. From Table 1, we see that unlike the case for oxygen, which promote *k*_*isc*_ and *k*_*T*_ to an equal extent, the spin labels enhance *k*_*isc*_ much more than *k*_*T*_. Furthermore, we observed a relative difference between the two quenchers, with TEMPO-PC acting as the better triplet state quencher and 16-DOXYL as the better intersystem crossing promoter. Both of these effects can likely be attributed to the fixed positioning of the quenchers (unlike freely diffusing oxygen), and their different locations in the membrane with respect to the NBD probe. The overall triplet quenching efficiency observed for a particular dye/quencher pair is thus dependent not only on their mobility, but also on the relative positions and orientations of the fluorophores versus the quenchers in the membrane.

To maximize the contrast in our readout, we investigated five different NBD-probes to determine the one with the best potential quenching efficiency in the membrane. The probes were based on PC and PE lipids labelled with NBD either on the sixth carbon (PC6, PE6), or on the 12th carbon (PC12, PE12) of one of their aliphatic tails (Fig. 3D, see Methods and Materials). We also tested a probe with NBD tagged on the 25^th^ position of cholesterol (NC). The quenching efficiency was defined as

1 $$\begin{array}{c}{\Phi }_{Q}=\frac{{A}_{TRAST}(0 \% )-{A}_{TRAST}(1 \% )}{{A}_{TRAST}(0 \% )}\end{array}$$

and represents the normalized difference in the overall decay amplitudes of the TRAST curves, recorded with and without 1% molar fraction of quencher added to the membrane. *A*_*TRAST*_ is the overall fraction of dark state NBD fluorophores generated upon long (1 ms) excitation pulses. The TRAST measurements described above for NBD-PC12 (Fig. 3A,B) were then repeated for the other dye/quencher pairs. The observed Φ_*Q*_ values are presented in Fig. 3C. These results suggest that of the probes tested, the best contrast is obtained with the spin labels acting on either NBD-PC12 or NBD-PE12. The lowest contrasts were seen for the PC6 and PE6 probes, which interestingly showed equally low sensitivity to quenchers located at the membrane surface (TEMPO-PC) and deep into the membrane (16-DOXYL).

### Lipid-lipid interactions in living cells

**Figure 4:**
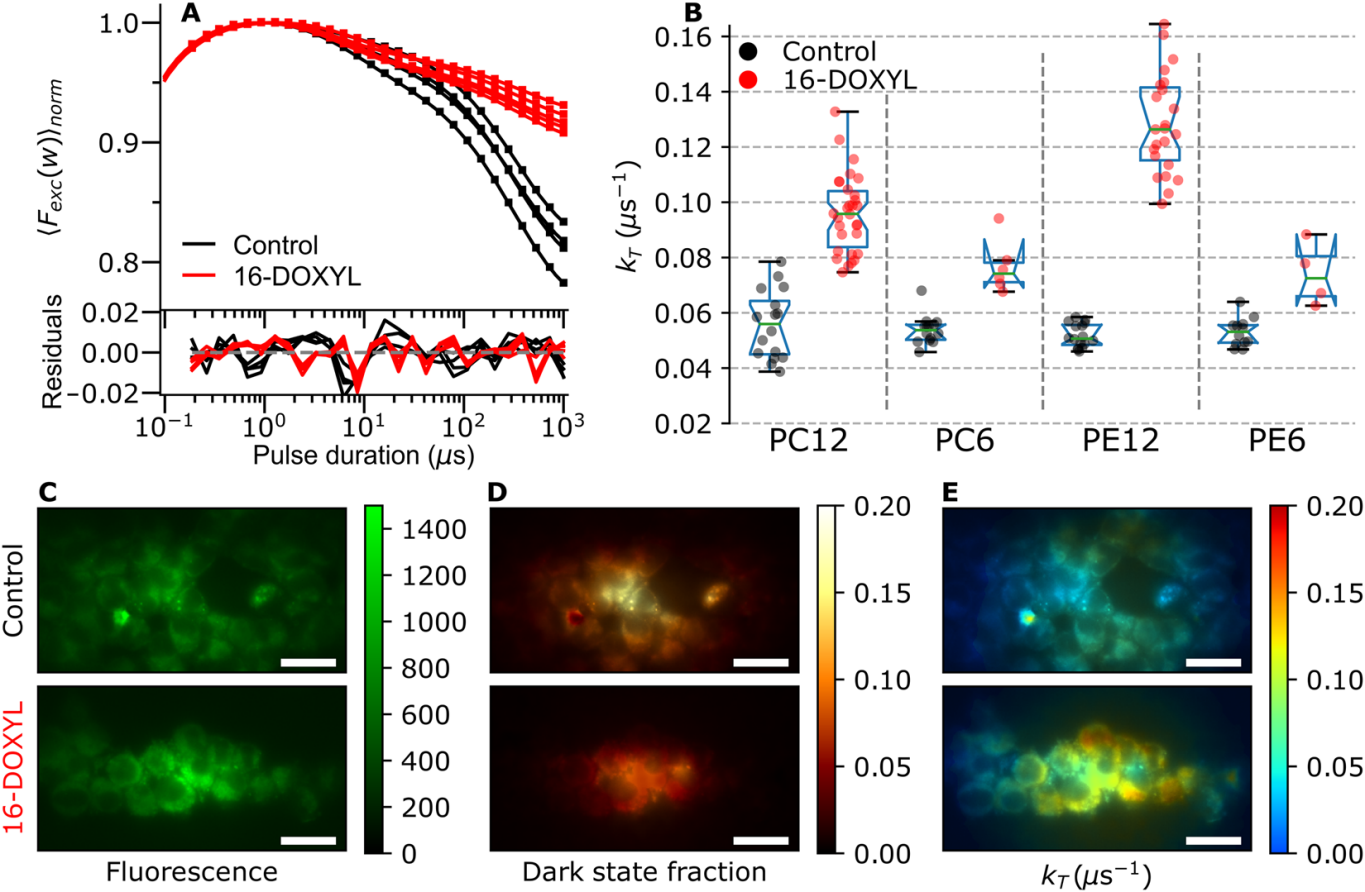
Live cell measurements from 293 T cells in PBS and deoxygenated atmosphere. Mean excitation irradiance rate used: 0.48 kW/cm^2^ **(A)** TRAST curves from NBD-PE12, with and without 1% molar fraction of 16-DOXYL added to the membrane. The TRAST curves were calculated for individual cells, based on the images in C. **(B)** Fitted triplet relaxation rates for different NBD probe configurations, with and without 1% 16-DOXYL. The *k*_*T*_ rate has been fitted from TRAST curves such as in A. Each circular marker represents one individual cell. **(C)** Typical NBD fluorescence images before dark state build-up, *F*(*w*_*short*_). **(D)** Corresponding ratio images showing local dark state fractions, *A*_*TRAST*_ = (*F*(*w*_*short*_)−*F*(*w*_*long*_))/*F*(*w*_*short*_). Dark state populations are largest at the center of the excitation beam. **(E)** Dark state population converted to triplet relaxation rates according to the photophysical model in Fig. 2C. Scale bar is 20 *μm*. In the cellular images (**C–E**), a slight roundedness of the cells can be noted, likely attributed to the de-oxygenation prior to the measurements.

To extend our assay to cellular events, we tested the potential contrast of the same NBD-lipid probes in the membranes of 293 T cells. The NBD-lipids were added using a 1 µM solution of complex between the lipid and BSA, leading to an observable labelling of the cell membrane. For better control of the quencher concentration in the membrane, we opted for the 16-DOXYL quencher, which for the labeling can be added directly to the solution. We then used an estimation of 0.125 pmol of lipids per cell and a 90% partitioning of 16-DOXYL to the cell membrane^42^ to calculate the final quencher concentration. To maximize the contrast during probe evaluation, we completely de-oxygenated the measurement chamber for 40 minutes before measurements. TRAST curves were then acquired using the same wide-field setup as for the SUV experiments above. Figure 4A shows typical TRAST curves (calculated as whole-cell averages) measured from NBD-PE12 labelled cells. Addition of DOXYL-16, to an estimated quencher concentration of 1% molar fraction in the cell membrane, led to a significant decrease in the overall dark state fraction, *A*_*TRAST*_, for all cells in the field of view.

To quantify the extent of dark-state state quenching, whole-cell TRAST curves were fitted with *k*_*T*_ as the only free parameter. In the fit, *k*_*isc*_ was calculated based on the known relationship between *k*_*T*_ and *k*_*isc*_ from the vesicle measurements. Given the photophysical model of Fig. 2C, with photo-oxidation of NBD taking place solely from its triplet state, T, any effects on the redox rates by the spin labels were assumed to be minor, and to have a negligible influence on the overall build-up of dark-state NBD fluorophores. In the fittings, the redox rates could therefore be fixed to the values given in Table 1, and the overall interaction rate between NBD and the quencher molecules in the membrane could thus be entirely reflected in the fitted *k*_*T*_ values. Just as in SUVs, NBD was found to experience a much lower quencher interaction when bound to the sixth carbon of the aliphatic tails of the lipids (PE6, PC6). For NBD-PC6 in cells, the mean triplet relaxation rate, ${\bar{k}}_{T}$, increased from 0.054 µs^−1^ to 0.074 µs^−1^ upon quencher addition, and similarly for NBD-PE6, from 0.053 µs^−1^ to 0.072 µs^−1^ (Fig. 4B). In comparison, for NBD-PC12 we observed an increase in ${\bar{k}}_{T}$ from 0.056 µs^−1^ to 0.096 µs^−1^ for the same concentration of quencher in the membrane, while for NBD-PE12 the change was 0.051 µs^−1^ to 0.126 µs^−1^ (Fig. 4B). Overall, the *k*_*T*_ quenching by 16-DOXYL is about half as efficient in cells when compared to SUVs, suggesting either a lower mobility of these probes in the cell membrane, or a significant internalization or neutralization of these spin-labels by the cell. We also found a clear cell-to-cell variability, possibly due to uncertainties in the added quencher concentrations. The most efficient lipid probe in cells turned out to be NBD-PE12, unlike in POPC SUVs where NBD-PC12 generated a somewhat higher increase of ${\bar{k}}_{T}$.

### Conversion of regular fluorescence images of living cells into TRAST images

In addition to analyzing the wide-field image data as cell averages, we computed the pixel-by-pixel dark state fractions, *A*_*TRAST*_, from the ratio between the average fluorescence intensity acquired within excitation pulses of close to 1 μs and 1 ms, respectively (see Methods and Materials). In contrast to regular fluorescence intensity images of NBD-PE12, which do not reveal any particular effect of quencher addition (Fig. 4C), the corresponding TRAST images show an overall reduction in dark state population of NBD in cells with quencher added to the membrane. However, Fig. 4D also reveals a strong dependence on excitation irradiance (as seen in Fig. 2A), with the largest dark state fractions in the center of the Gaussian excitation beam. To remove this excitation dependence and make it possible to compare with the whole-cell average recordings (Fig. 4B), we also calculated the pixel-by-pixel *k*_*T*_ rates (Fig. 4E). To reduce computational time when processing a large number of pixels, we used a pre-computed conversion table, directly relating local excitation irradiance and dark state population to *k*_*T*_ (see Methods and Materials). Once such a mapping has been computed, based on a known quenching model (Fig. 2C) and reference measurements in cells (Fig. 4A), a pair of regular fluorescence images (recorded with fast and slow excitation pulse durations) can be directly converted into a *k*_*T*_ rate image, in real time. In this way we could image local variations in the *k*_*T*_ rate in live cells, directly representing NBD-PE12 and 16-DOXYL encounter frequencies at subcellular resolution.

### Lipid-receptor interactions in cells

**Figure 5:**
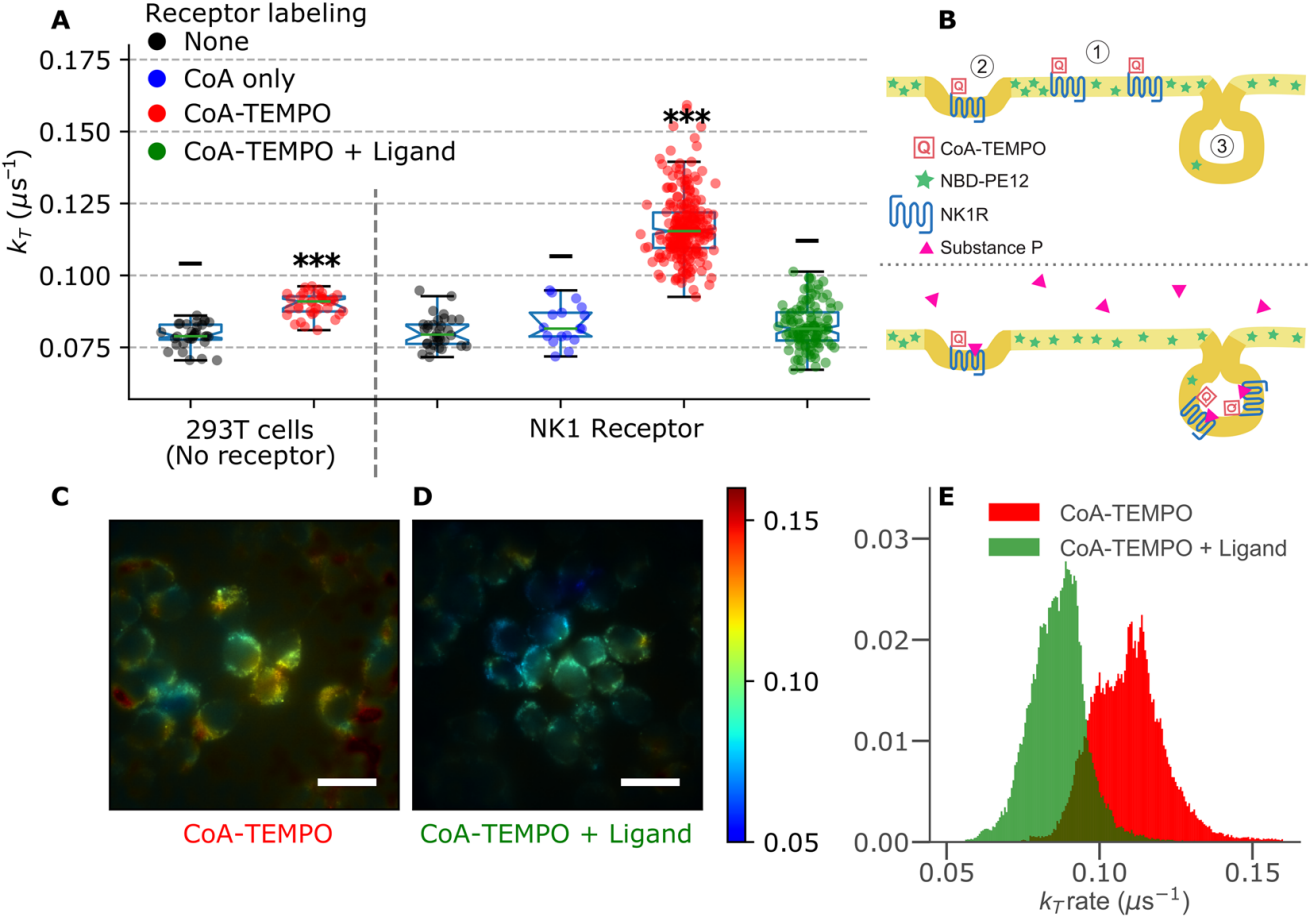
Determination of the collision frequency between receptors and lipids in cells. Measured in 2% oxygen atmosphere. **(A)** Fitted triplet relaxation rates of NBD-PE12, with and without CoA-TEMPO on the NK1 receptor. Each circle represents one cell. **(B)** Illustration showing probe and quencher locations, upon receptor activation. NK1R is found in distinct membrane domains (1 and 2) with potentially different lipid compositions. Activation of the receptor with its natural ligand SP triggers internalization and decreases the collisions rate between the NBD-labelled lipids and the quenchers bound to the receptors. TRAST images showing pixel-wise triplet relaxation rates in control cells **(C)** and in ligand activated cells **(D)**. Scale bar is 20 *μm*. **(E)** Pixel distributions of triplet relaxation rates for the images in C and D. Mean excitation irradiance rate used: 0.48 kW/cm^2^.

To assess the ability to observe collisional interactions between membrane proteins and lipids in membranes of living cells, we used a cell line stably expressing a fusion protein of NK1R and an ACP-tag. This tag allows a specific and stochiometric labelling of the receptor expressed in the cell membrane. The concentration of receptors in the plasma membranes was determined to ~2500 μm^−2^ by FCS measurements, see SI section 4. We then labelled NK1R with CoenzymeA-TEMPO and inserted NBD-PE12 in the membrane using 1 µM of a BSA/NBD-lipid complex (Fig. 5B, upper). NBD-PE12 is assumed to remain in the outer leaflet of the cell membrane for the duration of the measurements (<90 min), such that it is accessible for quenching by the receptor-bound TEMPO. Any NBD-PE12 flipping to the inner leaflet would lower the average dye/quencher interaction rate and lead to an underestimation of ${\bar{k}}_{T}$. Whole-cell TRAST measurements were performed as above, on cells in an atmosphere of 2% oxygen to preserve their viability. As for the lipid-lipid interaction measurements (Fig. 4B), the TRAST curves were fitted with *k*_*T*_ as the only free parameter. The outcome of the analyses are shown in Fig. 5A; Labeling of CoA-TEMPO on the NK1R-receptors was found to significantly increase *k*_*T*_ of NBD-PE12 freely diffusing in the cell membrane, from ${\bar{k}}_{T}$ = 0.080 μs^−1^ in the absence of quencher to ${\bar{k}}_{T}$ = 0.12 μs^−1^. Labeling the same receptor with only CoA, but without TEMPO attached, showed no significant effects on *k*_*T*_ (Fig. 5A, blue). This demonstrates that *k*_*T*_ specifically reflects the diffusional encounter rate between PE12 lipids and NK1R GPCRs in the cell membrane. As for the lipid-lipid interaction measurements (Fig. 4B), there is a broad distribution of *k*_*T*_ values for NBD-PE12 quenched by NK1R-TEMPO. Since the labelling efficiency of TEMPO to the receptors should not vary significantly between cells, this variability rather suggests a large cell-to-cell variability in the GPCR-lipid interactions, or in the density of the receptors in the cell membranes.

As a negative control we also performed the corresponding experiment in 293 T cells, which do not express NK1R (Fig. 5A). When repeating the same CoA-TEMPO labeling procedure for these cells, we observed a statistically significant increase, from ${\bar{k}}_{T}$ = 0.079 μs^−1^ to ${\bar{k}}_{T}$ = 0.089 μs^−1^, indicating that there is some non-specific binding of CoA-TEMPO. However, this increase in *k*_*T*_ was considerably smaller than that observed with specific NK1R receptor labelling.

In comparison to the previous SUV measurements, 2500 NK1R-TEMPO per μm^2^ would correspond to a quencher concentration of [TEMPO] = 0.19 mol percent in the case of a pure lipid bilayer (assuming one lipid occupies 0.75 nm^2^)^43^. A corresponding *k*_*T*_ quenching coefficient for NK1R-TEMPO can then be estimated to Δ${\bar{k}}_{T}$ = 0.21μs^−1^ (mol%)^−1^ [TEMPO] This quenching coefficient is almost identical to that found for NBD-PC12/TEMPO-PC in vesicles (0.22 μs^−1^ (mol%)^−1^, see Fig. 3B and Table 1). Since the diffusion coefficient of NK1R in the cellular membranes can be expected to be more than an order of magnitude lower than that of diffusing lipids in SUVs, one would expect the dye/quencher interaction rate to be lowered by almost a factor of two when TEMPO is placed on NK1R, as compared to when placed on POPC in the SUVs (assuming the diffusion coefficient of the lipids to be the same in the SUVs and in the cells). In agreement with this, if contributions from non-specific binding, as seen in Fig. 5A, is subtracted a lower Δ${\bar{k}}_{T}$ of 0.16 μs^−1^ (mol%)^−1^ is obtained. Additional factors may also compensate for the lower diffusivity of NK1R. In the cells studied, the quenching coefficient showed variability, both in the studied lipid-lipid and lipid-protein interactions (see Figs. 4B and 5A). This variability can have several reasons; The composition of the plasma membrane is heterogeneous. As a consequence, the local fluidity in the vicinity of the receptors can be expected to vary and may also influence the preference for PE12 to localize near NK1R. The mutual orientation and position of the TEMPO spin label quenchers versus the NBD fluorophores can also be different. Additionally, an uneven distribution of NK1Rs in the cellular membranes would lead to a variability in the effective quencher concentration experienced by the fluorophore-labeled lipids.

### Receptor activation

Addition of substance P (SP) is known to trigger receptor internalization and to significantly modify the surface distribution of NK1R^33,34^. The internalization process of the NK1R-proteins after activation efficiently decreases the amount of freely diffusing receptors, thereby reducing the number of TEMPO spin-labels in the membrane (Fig. 5B, lower). We used our TRAST sensor to quantify the effect of NK1R-activation, reflected via a change in the collisional quenching rate of the triplet state of NBD-PE12 by NK1R-TEMPO. Activation of the receptor with 0.5 nM of SP was found to significantly decrease the triplet decay rate, from ${\bar{k}}_{T}$ = 0.12 μs^−1^ in absence of ligand, to ${\bar{k}}_{T}$ = 0.082 μs^−1^ upon addition of SP, see Fig. 5A (green). The *k*_*T*_-values upon SP activation are comparable to the triplet relaxation previously seen in absence of quencher, indicating an efficient separation of NK1R from PE12 in the membrane, possibly by removal of the receptor from the plasma membrane by internalization.

We also performed TRAST imaging to visualize the effect of NK1R receptor activation. Figures 5C,D show *k*_*T*_-images, produced as previously described (Fig. 4E), for cells labelled with NBD-PE12 and CoA-TEMPO tagged on the NK1R receptors. Before activation with substance P, there is a significant variability in the *k*_*T*_ values, both within cells and between different cells (Fig. 5C), suggesting a complex diffusional map throughout the cultured cells. After activation, the cell-to-cell variability decreases, and a lowered, more homogenous distribution of triplet quenching is observed in the cells (Fig. 5D). Histograms of the *k*_*T*_ values of the imaged cells in Fig. 5C,D are shown in Fig. 5E. These distributions are both very similar to the corresponding ones in Fig. 5A, indicating that changes in the transient encounters of NK1R- TEMPO and NBD-lipids upon ligand activation can be clearly resolved by sub-cellular analysis of only a handful of cells.

## Discussion

In this study we present an experimental approach, combining the detection sensitivity of the fluorescence signal from NBD fluorophores with the environmental sensitivity of the long-lived dark transient states of the same fluorophores. Thereby, it is possible to follow low-frequency collisional encounters between lipids and proteins in artificial membranes and in the membranes of living cells. This approach fills an important niche; Transient collisional interactions between lipids and membrane proteins play an important role in modulating cellular functions, but occur at too low frequencies to be readily observable via fluorescence imaging or quenching studies. TRAST imaging, used to monitor the triplet state quenching of NBD fluorophores by spin labels in the membranes, offers a robust and widely applicable approach for such studies. Both NBD and the spin labels used are small and are added in relatively low concentrations (typically ~1% or less). They are therefore less likely to interfere with major cellular functions, with the membrane itself or with interactions within the membrane during the time of measurement.

In this work, we first established a photodynamic model of NBD, then studied the NBD dark-state transitions in SUVs and live cells for different NBD-lipid derivatives and spin labels. We then showed that changes in the triplet state decay rate, *k*_*T*_, directly reflects the collisional interaction rates between specific molecules in the membranes. To enhance the relative quenching effects of the spin labels on the NBD triplet state and minimize contributions from oxygen, the measurements were performed at deoxygenated or lowered oxygen concentrations. However, the influence of spin labels on the triplet state kinetics of dyes can vary considerably from one dye to another^24^. For other dye-spin label combinations, with relative quenching effects less influenced by oxygen, significant effects on the triplet state kinetics can also be expected to be well observable in air-saturated samples. *k*_*T*_ can be determined either on a whole cell level, and also on moving entities, such as the SUVs in this work. Alternatively, *k*_*T*_ can also be imaged in surface attached cells, thereby providing direct imaging of membrane mobility with subcellular resolution. The measured *k*_*T*_ on the one hand reflects the interactions between specific (NBD- and spin-labeled) molecules in the membranes. The quenching efficiency of NBD triplet states by the spin labels depends on the lipid head group and on the position of the NBD probe in the membrane. On the other hand, using the combination of NBD and spin labels as a bimolecular membrane mobility probe also senses the overall membrane fluidity and organization, where e.g. the slower diffusion coefficients and the complex environment in cellular plasma membranes decrease the lipid-quencher collisional rates, compared to in the membranes of the SUVs. In this study, we showed that ligand-dependent GPCR activation led to specific changes in *k*_*T*_, reflecting the induced reorganization of the local membrane environment, including internalization/removal of GPCRs from the plasma membrane. These changes in *k*_*T*_ coincided well with known activating ligand concentrations for the canonical pathway of the specific GPCR-ligand pair studied (NK1R and SP) and were observed independently of the receptor downstream signaling pathway. This shows that the presented approach offers a new strategy to monitor ligand-activation of GPCRs, via changes in *k*_*T*_. Notably, in this activation assay, the ligands themselves need not to be labeled. In a more general case, also the membrane receptors do not necessarily have to be labeled, as long as the ligand activation results in an overall change in the membrane organization and/or fluidity.

## Methods and Materials

### TRAST spectroscopy

TRAST monitors fluorophore blinking kinetics, not by resolving individual blinking events, but by monitoring the reduction in average fluorescence intensity from a large population of independently blinking fluorophores. Using modulated excitation, systematically varied on the time scales of the fluorophore dark-state to be studied, rapid blinking kinetics can be quantified without the need for time-resolved detection. This enables wide-field cellular imaging of μs blinking kinetics, using a regular camera and an exposure time of seconds.

Given the photophysical model for NBD in Fig. 2C, it is straightforward to simulate the instantaneous fluorescence signal, *F*(*t*), from a solution sample (see SI and Eq. S6). The typical behavior of *F*(*t*) is to show characteristic decays on a μs to ms time scale, as long-lived fluorophore dark states become populated, see Eq. (S5). A similar decay can also be observed in the time-averaged fluorescence signal resulting from a rectangular excitation pulse of duration *w*

2 $$\begin{array}{c}\langle {F}_{exc}(w)\rangle =\frac{1}{w}{\int }_{0}^{w}\,F(t)dt,\end{array}$$

as *w* is varied in the μs to ms time range. This is the basis for TRAST monitoring.

To obtain sufficient photon counts, even for short *w*, we collect the total signal resulting from an excitation pulse train of N identical pulse repetitions. The number of pulses is adjusted to maintain a constant laser illumination time, *t*_*ill*_ = *N*⋅*w* = 10 ms, for all *w*. A so-called TRAST curve is then calculated from the time-averaged fluorescence signal during excitation for each pulse train, normalized with the average signal at a given pulse duration, *w*_0_

3 $$\begin{array}{c}{\langle {F}_{exc}(w)\rangle }_{norm}=(\frac{1}{N}\mathop{\sum }\limits_{i=1}^{N}\,{\langle {F}_{exc}(w)\rangle }_{i})/(\frac{1}{{N}_{0}}\mathop{\sum }\limits_{i=1}^{{N}_{0}}\,{\langle {F}_{exc}({w}_{0})\rangle }_{i})\end{array}$$

The pulse duration used for normalization, *w*_0_, is chosen to be the *w* producing the highest fluorescence signal. Since the rise time of *F*(*t*) upon onset of excitation is in the nanosecond time range, *w*_0_ should be short enough (typically sub-μs) not to lead to any noticeable build-up of dark transient states.

In the above expression, 〈*F*_*exc*_(*w*)〉_*i*_ represents the total signal collected from the i:th pulse in the pulse train, as defined in Eq. (2). By using a low excitation duty cycle, here *η* = 0.01, fluorophores are allowed to fully recover back to S_0_ before the onset of the next pulse, making all pulses in a given pulse train identical. The summations in Eq. (3) are then no longer required and the expression simplifies further. Also note that in the normalization step of Eq. (3), several constants used to calculate *F*(*t*) in Eq. (S6) cancel out. The final expression for 〈*F*_*exc*_(*w*)〉_*norm*_ therefore becomes independent of fluorophore concentration, S_1_ decay rate, fluorescence quantum yield, and overall detection quantum yield of the microscope.

A complete TRAST experiment consisted of a stack of 30 fluorescence images, measured in rapid succession over a total time of less than 1 minute. Each image represents the total fluorescence signal from an entire excitation pulse train, captured using a camera exposure time of *t*_*exp*_ = *t*_*ill*_/*η* = 1 s. Pulse durations, *w*, were distributed logarithmically between 100 ns and 1 ms and measured in a randomized order to avoid bias due to time effects. An additional 10 reference frames, all using 100 ns pulse duration to avoid dark state build-up, were inserted at regular intervals between the 30 main images to track any permanent bleaching of the sample.

### Instrumentation

TRAST measurements were performed on a standard, inverted epi-fluorescence microscope (Olympus, IX73), with a 488 nm diode laser as excitation source (Cobolt, 06-MLD, 488 nm, 200 mW, Semrock BrightLine 488/10 excitation filter). The laser beam was modulated by an acousto-optic modulator (AOM; AA Opto Electronic, MQ180-A0,25-VIS), and focused close to the back aperture of the objective (Olympus, UPLSAPO 60x/1.20 W) to produce wide-field illumination in the sample. The beam waist in the sample plane was 35 μm (1/*e*^2^ radius). The fluorescence signal, collected by the same objective, was separated from the excitation light by a dichroic beamsplitter (FF506-Di03, Semrock) and then fed to the microscope’s camera port. For detection, an sCMOS camera (Hamamatsu ORCA-Flash4.0 v2) was used with double emission filters (BrightLine 530/55, Semrock) to remove scattered laser light.

The experiments were controlled and synchronized by custom software implemented in Matlab. A digital I/O card (PCI-6602, National Instruments) was used to trigger the camera and generate excitation pulse train signals sent to the AOM driver unit.

For experiments with modified atmospheres, a stage incubator system (WP and FC-7, Chamlide, Live Cell Instruments) was used to control oxygen concentration.

### TRAST data analysis

The TRAST data was analyzed using software implemented in Matlab. The recorded TRAST images were first pre-processed by subtraction of the static ambient background, optional binning to either larger pixels or regions of interest (ROIs), and correction for bleaching. The bleaching correction was based on 10 reference frames, recorded in between the regular frames throughout the measurements (see TRAST spectroscopy above), and then interpolated into pixel-by-pixel bleaching curves. Based on these bleaching reference curves, correction for bleaching could be performed for each pixel in each frame. In the SUV measurements, the bleaching was partly counteracted by the diffusion of new vesicles into the detection area. The overall bleaching was then maximally 5–10% of the total detected intensity. For cells, where fluorophores were not replenished by diffusion, the bleaching was higher, in rare cases as high as 50%.

For vesicle measurements (Figs. 2 and 3), TRAST curves were produced by calculating 〈*F*_*exc*_(*w*)〉_*norm*_ within a region of interest (ROI) corresponding to a 23 μm radius in the sample plane, centered on the excitation beam. By sub-sectioning this ROI into radial shells, 〈*F*_*exc*_(*w*)〉_*norm*_ resulting from a range of excitation irradiances can be analyzed from a single wide-field TRAST image (Fig. 2A). Whole-cell TRAST curves (Fig. 4A) were calculated from ROIs manually drawn around each cell in the image. In both cases, fitting of photophysical rate parameters was then performed by simulating theoretical TRAST curves using Eqs. (S4, S6), (2) and (3) and comparing them to the experimental data. The set of rate parameters best describing the experimental data was then found using non-linear least squares optimization. In the fit, the *k*_10_ rate was fixed given the experimental lifetime of NBD (*τ*_*f*_ = 5.6 ns^36,37^, verified by time-correlated single photon counting measurements) such that 1/*τ*_*f*_ = *k*_10_ + *k*_*isc*_ in absence of spin-labels. The average observed excitation rate, ${\hat{k}}_{01}$, was calculated for each pixel or ROI using Eq. (S7) (see SI for details) and an excitation cross section for NBD of *σ* = 8.6 · 10^−17^ cm^2^ ^36,39^.

Images showing dark state fractions (*A*_*TRAST*_, Fig. 4D) were produced by calculating the normalized pixel-by-pixel (8 × 8 binning) difference in the recorded fluorescence intensity, between an image generated under short pulse duration, *F*(*w*_*short*_), compared to one generated under long pulse duration, *F*(*w*_*long*_).

4 $$\begin{array}{c}{A}_{TRAST}=\frac{F({w}_{short})-F({w}_{long})}{F({w}_{short})}\end{array}$$

To improve photon statistics, *F*(*w*_*short*_) was calculated as the sum of the intensities in 5 frames with pulse durations closest to 1 μs, while *F*(*w*_*long*_) was a combination of the 5 frames closest to 1 ms.

To convert images of *A*_*TRAST*_ (Fig. 4D) into *k*_*T*_ maps (Figs. 4E and 5C,D), a pre-computed conversion table was used. This table was constructed using the photophysical parameters of NBD, determined in vesicles (Figs. 2 and 3) and live cells (Fig. 4A,B). We thus considered both *k*_*ox*_ and *k*_*red*_ to be constants, unaffected by spin-labels. Based on the known quenching coefficients for *k*_*isc*_ and *k*_*T*_, we used this relation to calculate *k*_*isc*_ for each possible value of *k*_*T*_. The fitting could thus be reduced to a single free parameter, *k*_*T*_. Using the TRAST simulation techniques described above, we then constructed a conversion table relating the average excitation rate, ${\hat{k}}_{01}$ (given by Eq. S7), and dark state fraction, *A*_*TRAST*_, to the local *k*_*T*_ rate. Once such table has been computed for a particular fluorophore and set of experimental conditions, a large number of pixels can be quickly processed. Real-time conversion between a pair of fluorescence images (*F*(*w*_*short*_) and *F*(*w*_*long*_)) into a *k*_*T*_ image showing local dye/quencher interaction rates is then possible.

### Chemicals

POPC (*1-palmitoyl-2-oleoyl-glycero-3-phosphocholine*), TEMPO-PC (*1,2-dipalmitoyl-sn-glycero-3-phospho(tempo)choline*), NBD-PE12 (*1-oleoyl-2-[12-[(7-nitro-2-1,3-benzoxadiazol-4-yl)amino]dodecanoyl]-sn-glycero-3-phosphoethanolamine*), NBD-PC12 (*1-Oleoyl-2-[12-[(7-nitro-2-1,3-benzoxadiazol-4-yl)amino]dodecanoyl]-sn-Glycero-3-Phosphocholine*), NBD-PE6 (*1-oleoyl-2-[6-[(7-nitro-2-1,3-benzoxadiazol-4-yl)amino]hexanoyl]-sn-glycero-3-phosphoethanolamine*), NBD-PC6 (*1-oleoyl-2-[6-[(7-nitro-2-1,3-benzoxadiazol-4-yl)amino]hexanoyl]-sn-glycero-3-phosphocholine*) and NBD-Chol (*25-[N-[(7-nitro-2-1,3-benzoxadiazol-4-yl)methyl]amino]-27-norcholesterol*) were all purchased from Avanti Polar lipids. All other chemicals were purchased from Sigma-Aldrich.

### Liposome formation

Small unilamellar vesicles (SUV) were made by sonication. A chloroform solution of 1.4 mg of POPC, containing a fraction of fluorescent NBD-labeled lipids between 1:20000 and 1:5000, were dried under a gentle flow of N_2_ in a 4 ml Supelco glass vial. After complete CHCl_3_ evaporation at room temperature, 1.4 ml HNMG (10 mM HEPES, 150 mM NaCl, 5 mM MgCl_2_, 5% glycerol, pH 7.4) was added to the dried lipids. After 15 min at room temperature, the lipid suspension was vortexed until all large aggregates were removed. The suspension was then sonicated for 3 min, using a Branson SFX250 sonicator at 75% duty cycle (1.50 s on, 0.50 s off), 50% power (125 W) and a 1/8” microtip (Emerson Electric Co, St. Louis, MO, USA). To avoid overheating and evaporation of the solution, the bottom of the glass vial was kept in contact with ice-cold water. Sonication changed the lipid suspension into a typical glassy solution. This solution was centrifuged for 20 minutes at 15’000 g, and the supernatant then filtered on a 0.2 µm spin-filter (Corning, NY, USA) to remove potential large aggregates of lipids. This method produces SUVs with diameters of 25 to 30 nm, as verified by FCS measurements. Each vesicle therefore contained between 1 and 5 fluorophores. TEMPO-PC containing SUVs were made by adding 0.5 to 2 mol% of TEMPO-PC to the initial lipid mixture. When used, DOXYL stearic acid derivatives were added to the final SUV solution to reach final molar fractions between 0.25 and 4%.

### Lipid preparation for cell membrane labelling

Cell membranes were labelled with NBD lipid derivatives using a BSA-lipid complex according to the simplified protocol of Eggeling *et al*.^44^ 20 nmol of the lipid stock solutions in CHCl_3_ were dried under nitrogen. Dried lipids were dissolved in 4 μl of absolute ethanol and vortexed vigorously after addition of 200 μl of defatted BSA solution (100 μM BSA in DMEM/F12). Addition of 1.8 ml DMEM/F12 led to a 10 μM BSA and 0.2% ethanol solution.

### TEMPO-CoA synthesis

TEMPO-CoA was synthesized by reacting 4 mg 4-Maleimido-TEMPO (Sigma, 253359) with 2 mg Coenzyme A sodium salt hydrate (Sigma, C3144) in PBS (Sigma) for 2 hours at room temperature. TEMPO-CoA was purified overnight by dialysis at 4 °C on 500 Da membrane (Harvard Apparatus, USA).

### TRAST measurements of SUVs

For deoxygenated measurements, vesicle solutions were placed under 100% argon atmosphere for one hour prior to experiments to remove oxygen and were then kept under anoxic atmosphere during the whole experiment. Measurements with varying oxygen concentration were performed in a stage incubator on the microscope (see Instrumentation), in a controlled mixture of nitrogen and oxygen.

### Cell preparation

293 T cells stably expressing GPCRs (Tachykinin, NK1)^34^ were grown in DMEM/F12 medium supplemented with 10% fetal bovine serum (Life Technology), penicillin and streptomycin. Cells were sub-cultured 24–48 hours before TRAST experiments in glass-bottom 8-well plates (Nunc Lab-Tek II Chambered Coverglass).

Fluorescent lipids, containing NBD at different positions on their aliphatic chains were added as complexes with BSA, in cell growth medium on ice for 30 min. Cells were washed 5 times with ice-cold PBS after lipid insertion. Spin labels were added, either as a tag on the GPCRs, or in the membrane (DOXYL16-stearic acid). ACP- NK1 receptors were labelled with TEMPO-CoA according to supplier guidelines. Labelling was made with a solution containing 2 µM TEMPO-CoA, 1 µM of ACP-Synthase (New England Biolabs, USA) and 10 mM MgCl2 in DMEM/F12 supplemented with 10% FBS. Cells were incubated 30–40 minutes at 37 °C and then washed 5 times with ice cold PBS. The oxygen level was maintained at 2% during the whole duration of the experiments using a stage incubator on the microscope (see Instrumentation).

## Supplementary information

Supplementary info
