# Supplementary material for: Imaging of intermittent lipid-receptor interactions reflects changes in live cell membranes upon agonist-receptor binding
Source: Sci Rep. 2019 Dec 2;9:18133. doi: 10.1038/s41598-019-54625-w (PMC6889430; doi:10.1038/s41598-019-54625-w)
Supplement: Supplementary file 1 — Supplementary info [file 41598_2019_54625_MOESM1_ESM.pdf]

Supplementary Information

Imaging of intermittent lipid-receptor interactions reflects changes in live cell membranes upon agonist-receptor binding

Johan Tornmalm[1, #], Joachim Piguet[1, #], Volodymyr Chmyrov[1], Jerker Widengren[1]

[1]Experimental Biomolecular Physics, KTH, 10691, Stockholm, Sweden
# These authors contributed equally to this work
jpiguet@kth.se, jwideng@kth.se

1 ELECTRONIC STATE MODEL FOR NBD

Based on the NBD electronic state model shown in Figure 2C, the population dynamics of each state can be described by

$$\frac{d}{dt}\bar{P}(t) = M \cdot \bar{P}(t) \tag{S1}$$

, where $\bar{P}(t) = [S_0(t) \quad S_1(t) \quad T_1(t) \quad R_1(t)]^T$ represents the population probabilities of each electronic state at time t and

$$M = \begin{bmatrix} -k_{01} & k_{10} & k_T & k_{red} \\ k_{01} & (-k_{10} - k_{isc}) & 0 & 0 \\ 0 & k_{isc} & (-k_T - k_{ox}) & 0 \\ 0 & 0 & k_{ox} & -k_{red} \end{bmatrix} \tag{S2}$$

is the model matrix describing all transitions between those states. The initial condition for Eq. (S1) is

$$\bar{P}(0) = [1 \quad 0 \quad 0 \quad 0]^T \tag{S3}$$

, assuming all fluorophores are in the singlet ground state before the onset of excitation at $t = 0$.

The total S_1 decay rate is constrained by the experimental fluorescence lifetime, $k_{10} + k_{isc} = {}^1/_{\tau_F}$, while the excitation rate is calculated from $k_{01} = \sigma \cdot \Phi_{exc}$. Here σ is the fluorophore excitation cross section at the given wavelength and Φ_{exc} is the local excitation photon flux. For a rectangular excitation pulse, Φ_{exc} is constant throughout the excitation duration and the matrix M is not time dependent. The general solution to such a system of equations is then

$$\bar{P}(t) = e^{Mt} \cdot \bar{P}(0) \tag{S4}$$

While the expression in Eq. (S4) is normally evaluated numerically when fitting TRAST data, the analytical solution can be informative to understand the typical behavior of TRAST curves. Given the problem in (S1)-(S3), a general analytical expression for $S_1(t)$, the source of fluorescence photons, is

$$S_1(t) = \frac{k_{10}}{k_{10} + k_{10}}\left[1 - e^{-\lambda_{ab}t} - \sum_{i=1}^{n}\left[A_i - A_i e^{-\lambda_i t}\right]\right] \tag{S5}$$

Here n denotes the number of long-lived, photo-induced, non-fluorescent states in the model (for the model in Figure 2C and Eq. (S2) these states comprise T and $\dot{R}^+$, such that $n = 2$), λ_{ab} and λ_i are the non-trivial eigenvalues of M, representing rates of relaxation, and A_i are the corresponding amplitudes. The S_0/S_1 equilibration time, often referred to as the anti-bunching time, is given by $\tau_{ab} = 1/\lambda_{ab}$ and is usually well approximated by $\tau_{ab} = 1/(k_{01} + k_{10})$.

For most fluorophores, including NBD, all long-lived transient states can be considered non-luminescent, such that essentially all collected photons originate from the S_1 excited singlet state. In the case of a homogeneous solution sample, the instantaneous fluorescence signal detected is therefore

$$F(t) = cq_f q_D k_{10} \iiint \left(CEF(\bar{r}) \cdot S_1(\bar{r}, t)\right) dV \tag{S6}$$

, where $CEF(\bar{r})$ is the collection efficiency function of the detection system, q_D the overall detection quantum yield, c is the fluorophore concentration and q_f the fluorescence quantum yield. For a sample with multiple emissive states, the above equation is easily modified to include additional components of the state vector.

2 Spatial distribution of excitation rates, calculation of average rates

The Gaussian shape of the excitation beam means that the excitation photon flux, $\Phi_{exc}(\bar{r})$, is a function of position in the sample. As a consequence, a detailed TRAST analysis should include a spatial dependence to both excitation rate, $k_{01}(\bar{r})$, and the resulting electronic state populations, $\bar{S}(t,\bar{r})$. The total fluorescence signal on each pixel of the camera then becomes a convolution of $S_1(t,\bar{r})$ and the microscope collection efficiency function, $CEF(\bar{r})$, as shown in Eq. (S6). However, simulating the whole 3D sample volume, and computing the projected 2D image on the camera, becomes a costly operation when performed in each iteration of the fitting algorithm. While this procedure is possible, and sometimes required, we found that pre-computing the average observed excitation rate, $\hat{k}_{01}$, for each pixel or ROI to be analyzed, speeds up the fitting significantly, without appreciable loss of accuracy. The approximate $\hat{k}_{01}$ is computed once, before fitting starts, by weighting $k_{01}(\bar{r})$ by brightness and collection efficiency, $CEF(\bar{r})$, in the following manner

$$\hat{k}_{01} = \frac{\iiint k_{01}(\bar{r}) \cdot \hat{S}_1(\bar{r}) \cdot CEF(\bar{r})\, dV}{\iiint \hat{S}_1(\bar{r}) \cdot CEF(\bar{r})\, dV} \tag{S7}$$

Here $\hat{S}_1(\bar{r}) = k_{01}(\bar{r})/(k_{10} + k_{01}(\bar{r}))$ is the S_1 population at onset of excitation, after equilibration of the singlet states, but before dark state build-up.

3 Quenching of the NBD triplet state by Doxyl-16

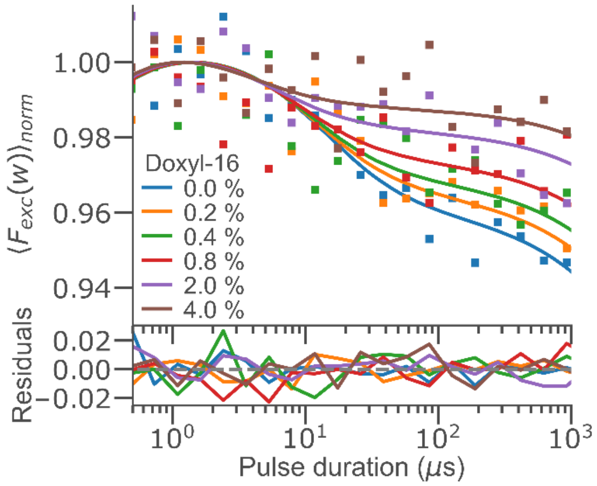

Supplementary Figure S1: *TRAST curves of NBD-PC12 in POPC vesicles with varying molar fractions of 16-Doxyl. The fitted triplet relaxation rates, k_T, are shown in Figure 3 and the corresponding bimolecular quenching coefficient in Table 1.*

4 MEASUREMENT OF THE RECEPTOR DENSITY

The average surface density of NK1R receptors in the cell membranes was measured using fluorescence correlation spectroscopy (FCS). Receptors were labelled with Atto594. The average number of receptors within the detection volume was then fitted to $N = 343$, which given a detection volume of radius $\omega = 209 \pm 20$ nm corresponds to a receptor density of $N/(\pi\omega^2) \approx 2500\ \mu\text{m}^{-2}$.

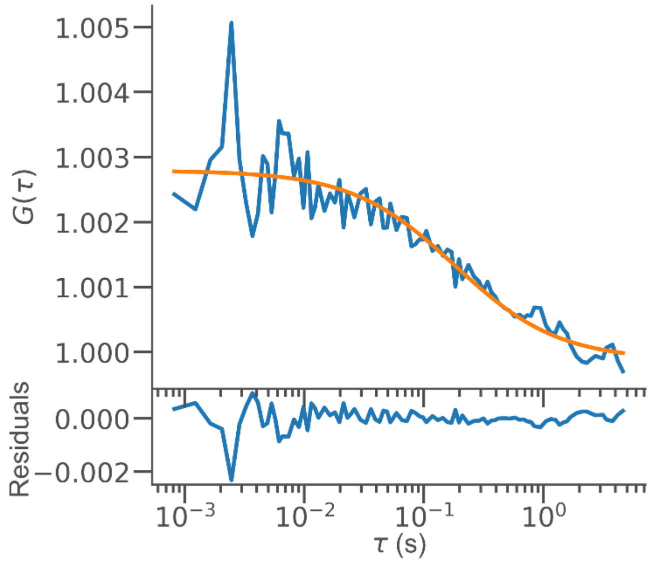

Supplementary Figure S2: FCS curve showing CoA-Att0594-labelled NK1-receptors diffusing in 293T cell membranes. The average number of receptors within the detection volume was fitted to $N = 343$, corresponding to a receptor density of $2500\ \mu\text{m}^{-2}$ in the membrane.

Fluorescent label synthesis

Atto594-CoA was synthesized by reacting 0.1 mg Atto594-maleimide (Atto-Tec, AD 594-41) with 0.05 mg Coenzyme A sodium salt hydrate (Sigma, C3144) in PBS (Sigma) for 2 hours at room temperature. The product was purified by dialysis at 4°C on 1kDa membrane (Harvard Apparatus, USA). NK1-receptors wer labelled following the procedure described in the main article.

FCS Instrumentation

FCS curves were acquired using an Abberior Instruments setup, built on an Olympus IX83 stand. Excitation was provided by an Abberior Instruments fiber-coupled diode laser, emitting at 594 nm and pulsed at 20 MHz, focused into the sample by a Leica 100x NA 1.4 objective. Fluorescence was collected through the same objective, directed to a motorized pinhole (MPH16, Thorlabs) and filtered with a FF01-615/20 (Semrock) filter. The signal was then detected by a single photon detector (SPCM-AQRH-13; Excelitas Technologies) and recorded by the Imspector software (Abberior Instruments). The confocal detection volume was calibrated to $\omega = 209 \pm 20$ nm $1/e^2$ radius.

FCS Analysis

A fluorescence time trace was correlated using a multiple-tau python module (*Paul Müller, 2012, Python multiple-tau algorithm, version 0.1.9, https://pypi.python.org/pypi/multipletau/*). The resulting auto-correlation curve was fitted (non-linear least squares) in Python using

$$G(\tau) = \frac{1}{N}(1 + \tau/\tau_D)^{-1} + offset$$

Here N is the average number of fluorophores within the detection volume and τ_D is the average diffusion time through the detection volume.
